# Single-dose intravenous ketamine or intramuscular naltrexone for high-utilization inpatients with alcohol use disorder: pilot trial feasibility and readmission rates

**DOI:** 10.1186/s13722-022-00345-y

**Published:** 2022-11-22

**Authors:** Dale Terasaki, Ryan Loh, Anastasia Cornell, Julie Taub, Christian Thurstone

**Affiliations:** 1 Department of Behavioral Health, Denver Health & Hospital Association, 777 Bannock St, Denver, CO 80204 USA; 2 Department of Medicine, University of Colorado School of Medicine, Aurora, CO USA; 3 Rocky Mountain Poison and Drug Safety, Denver, CO USA; 4 Division of Hospital Medicine, Denver Health & Hospital Association, Denver, USA; 5 Department of Psychiatry, University of Colorado School of Medicine, Aurora, CO USA

**Keywords:** Hospital utilization, Medication adherence, Alcohol use disorder, Ketamine, Naltrexone

## Abstract

**Background:**

Alcohol use disorder (AUD) accounts for millions of acute care encounters annually in the United States. Hospitalization represents a vital opportunity to intervene pharmacologically, but low medication adherence is a significant barrier. Two single-dose, adherence-independent interventions are well suited for pre-discharge administration: intravenous (IV) ketamine and intramuscular (IM) naltrexone. Their feasibility and readmission-reducing efficacy in hospital settings are not well-established.

**Methods:**

A 3-arm, open-label randomized trial was conducted at our safety-net medical hospital among high-utilization inpatients with severe AUD. Consented adults (age 18–65) were randomized to (1) IV ketamine (KET) 0.5 mg/kg over 40 min, (2) IM naltrexone (NTX) 380 mg once, or (3) linkage alone (LA). The primary clinical outcome was 30-day all-cause hospital readmission rate. All were provided enhanced linkage to outpatient addiction clinic.

**Results:**

We consented and randomized 44 participants (n = 13, 14, 17 for KET, NTX, LA, respectively), with a mean of 3.2 past-year hospitalizations. Compared to the LA arm, both the KET arm (RR 0.37, p = 0.17) and NTX arm (RR 0.52, p = 0.27) had a lower 30-day readmission rate, though the differences were nonsignificant. Immediate acceptability ratings of KET and NTX were 9.50 and 9.17 out of 10, respectively. No serious adverse events or illicit ketamine use was reported.

**Conclusions:**

Both interventions are feasible and showed promise in reducing readmissions for high-utilization AUD inpatients. Despite randomization, baseline characteristics may have differed in ways that biased against the control arm. Additional pragmatic studies—with larger sample size, blinding, and robust follow-up data collection—are needed to verify findings and better understand mediating factors.

*ClinicalTrials.gov Identifier* NCT04562779. Registered 24 September 2020. https://clinicaltrials.gov/ct2/show/NCT04562779

## Introduction

Alcohol use disorder (AUD) affected 14.5 million U.S. adults in 2019 [1], and national surveys indicate that alcohol-related problems and mortality increased substantially amid the COVID-19 pandemic [2, 3]. There are approximately 5 million U.S. emergency department (ED) visits every year related to alcohol [4], 40% of which result in hospital admission [4]. Because AUD treatment is seldom accessed [5], hospital admission represents a major opportunity for linkage to care [6, 7] and initiation of medications [8], but low treatment adherence and low self-efficacy undermine the potential benefits of AUD pharmacotherapy [9–11] in this population prone to readmission [12]. When administered as a single dose prior to discharge, two medications with prolonged effects may be well suited to reduce relapses that lead to readmissions: intravenous (IV) ketamine and intramuscular (IM) naltrexone.

Ketamine is an *N*-methyl-d-aspartate (NMDA) receptor antagonist used in general anesthesia and pain management. At sub-anesthetic doses, ketamine provides rapid effects on mood that extend days-to-weeks beyond administration [13, 14], thought to occur in part through modulation of synaptic plasticity, neurogenesis, and neural network connectivity [15]. Ketamine may be particularly well-tolerated among patients with physiologic alcohol tolerance, lacking the dysphoric symptoms that can be experienced by healthy controls [9]. In chronic alcohol consumption, NMDA receptors are upregulated, and changes in synaptic plasticity occur in key brain regions involved in addiction [16]. Ketamine’s anti-NMDA activity may disrupt these pathologic changes [15] and rapidly attenuate associations and memories [17] that reinforce drinking behavior.

Naltrexone is an antagonist at the mu-opioid receptor as well as the kappa- and delta-opioid receptors to a lesser extent [18]. Conventionally prescribed as a tablet taken by mouth (PO), it is a first-line AUD medication shown to attenuate the reinforcing effect of alcohol among heavy drinkers [19], improve a number of drinking outcomes [20], and reduce acute care utilization [8, 21]. Studies on IM naltrexone—which releases over 4 weeks after a transient initial peak—have shown better long-term medication continuation compared to PO naltrexone [22] and fewer heavy drinking days compared to placebo [23]. To date, there are few published data on initiation of PO [24] or IM [25] naltrexone before hospital discharge.

To further inform their use in the medical hospital setting, we sought to (1) test the feasibility and (2) estimate the readmission-reducing efficacy of a single IV ketamine infusion or IM naltrexone injection among high-utilization, medically hospitalized patients with AUD at our safety-net institution.

## Methods

An open-label, pragmatic, pilot randomized trial was conducted from January 2021 through December 2021. Each participant was randomly assigned in parallel to (1) IV ketamine (KET), (2) IM naltrexone (NTX), or (3) linkage alone (LA). All arms received enhanced linkage to our outpatient addiction clinic for continued AUD support. The primary clinical outcome was 30-day, all-cause, hospital readmission rate.

This study occurred at an urban, academic, safety-net hospital. Participants were recruited from the census of the addiction consultation service (ACS), a physician-led team that assists with evaluation, withdrawal management, pharmacotherapy, psychotherapy, care linkage, and harm reduction [26]. Enrollment, baseline data collection, and intervention were completed at bedside during participants’ index admission. Some follow-up data were collected at the outpatient addiction clinic visit if they presented. Follow-up attendance and readmission data were obtained through electronic health record (EHR) query.

This trial was approved by the Colorado Multiple Institutional Review Board.

### Selection of participants

To target those with high care utilization and ready access to our outpatient services, adult patients (age 18–65) with severe alcohol use disorder (six or more Diagnostic and Statistical Manual-V criteria [27]) were approached if they had one or more alcohol-related hospital admission(s) or emergency department (ED) visit(s) in the past year, had public (e.g., Medicaid) or private insurance, and were seen by the ACS. Exclusion criteria were: active COVID-19, being too medically ill for the interventions (AST/ALT > 5 × times upper-limit of normal, decompensated cirrhosis, glomerular filtration rate < 30 ml/minute, current/past acute coronary syndrome, cerebrovascular event, hypertensive crisis, cardiomyopathy, known elevated intracranial pressure, or platelets < 50/microliter), unresolved moderate/severe alcohol withdrawal, active delirium, active enrollment in another study, past-month receipt of IM naltrexone or IV ketamine, study medication intolerance, other substance use disorder (besides tobacco/cannabis), known/anticipated pregnancy or breast-feeding status, chronic/anticipated opioid use, unstable psychiatric illness (active psychosis or suicidality), and discharge to acute/residential treatment.

### Study procedures

For appropriately identified patients, recruitment occurred by the principal investigator after their initial addiction consultation. Consented participants were immediately assigned to one of the three arms through a simple randomization feature in the EHR. Participants provided baseline data, completed their clinic intake including appointment scheduling (targeted within 7 days post-discharge), and received their assigned intervention at bedside before discharge. To better isolate the effects of the pharmacological interventions, inpatient teams were instructed not to prescribe oral naltrexone (or acamprosate or disulfiram) on discharge, although participants could receive any medication at follow-up if appropriate. Notably, prescribing discharge medications for AUD is rare; a recent systematic review [24] found just two studies implementing naltrexone prescribing for AUD on discharge. Their baseline prescribing rates were 0.0 and 1.6%. Additionally, evidence showing effectiveness of in-hospital prescribing of AUD medications is scarce [24]. Therefore we did not consider lack of oral AUD medication prescription on discharge to be an egregious departure from standard of care.

Acceptability data were collected 40 min after the start of pharmacologic intervention. Stipends were provided for enrolling ($10) and presenting to follow-up appointment ($20).

For the KET arm, the intravenous infusion (at a dose commonly used in depression studies [14, 28], 0.5 mg/kg over 40 min) was administered by a registered nurse with the principal investigator at bedside on or near the day of anticipated discharge. The participant was monitored with telemetry and continuous pulse oximetry during the infusion and until at least 2 h after completion (160 min post-initiation). Vital signs were recorded at 0-, 40-, and 160-min post-initiation. The participant was provided an eye covering and noise-cancelling headphones that played a standardized, relaxing soundtrack [29]. The participant received brief pre-infusion counseling on what to expect, with specific advice to (1) focus on deep breathing and (2) approach difficult internal experiences with curiosity. Physical symptoms were assessed immediately before initiation and upon infusion completion (at 40 min). Dissociative symptoms were also assessed upon infusion completion.

For the NTX arm, the gluteal injection (380 mg) was administered by a registered nurse at bedside on or near the day of anticipated discharge. Nurse educators were trained in correct administration, and they subsequently supervised other nurses. Physical symptoms were assessed immediately before and immediately after injection. Vital signs were recorded at 0- and 40-min post-injection.

For the LA arm, no pharmacologic intervention was given as part of the study, but participants still received outpatient addiction clinic linkage and the research stipends. For comparison with other arms, baseline physical symptoms and vital signs were assessed on or near the day of anticipated discharge.

### Outcomes

The primary clinical outcome was all-cause, 30-day hospital readmission rate, assessed through EHR query that detects admissions to at least 34 other hospitals in the state (as of the start of the trial). This outcome was selected because: (1) it is highly relevant to clinicians [30], policymakers [31], and patients who often experience disease-related stigma in hospital settings [32, 33], and (2) it is not dependent on patient follow-up or recall, thus ensuring near-complete data collection among this inherently unstable population.

Primary feasibility outcomes included study recruitment rate, patient acceptability (10-point Likert scale), and adverse events. Main secondary clinical outcomes were 30-day all-cause emergency department (inclusive of our institution’s withdrawal management facility) visit rate and 14-day addiction clinic encounter (in-person or telehealth), ascertained through EHR query.

At baseline, self-reported daily drinking was recorded using a modified Timeline Follow Back (TLFB) [34] method (recording 7 days preceding admission). Adverse childhood events (ACE) were recorded using the 10-item ACE questionnaire [35]. Recent depressive symptoms were recorded using the Patient Health Questionnaire 9 (PHQ9) [36], a 9-item instrument commonly used for outpatient screening.

We assessed anticipated and perceived effectiveness of intervention in terms of reducing alcohol intake (10-point Likert scale) immediately following pharmacologic intervention and at follow-up, respectively.

We recorded immediate, treatment-emergent symptoms through a standardized, open-ended symptom inquiry (“Pay attention to your body. What symptoms are you noticing?”) assessed immediately pre- and post-pharmacologic intervention. Immediate dissociative symptoms from ketamine were recorded using the Clinician-Administered Dissociative States Scale (CADSS) [37], a 23-item questionnaire developed to measure dissociation due to acute stressors. For those that presented to their follow-up visit, adverse events since hospital discharge were recorded using the Patient Rated Inventory of Side Effects (PRISE) [38], a self-report, system-based (e.g. “cardiovascular”) questionnaire that assesses the presence and tolerability of symptoms.

### Statistical analysis

Data were analyzed between the LA arm and either KET or NTX arms. Descriptive statistics were used to analyze demographic variables, study feasibility, and acceptability. Hypothesis tests resulting in p-values for statistical significance were conducted judiciously; Table 1 (baseline characteristics, by arm) excludes p-values entirely because there is inherently no uncertainty that observed differences are based on random chance [39, 40]. When tested, continuous numerical data (e.g., vital signs) were compared via an appropriate independent or paired samples t-test. Proportional or count data (e.g., primary/secondary binary outcomes) were compared with a chi-squared test of independence, with effect sizes reported as relative risks. All reported p-values were considered significant at an alpha of 5%. In Table 1, missing values were included in “other” or “unknown” for all self-reported demographics, except for family history (considered negative if missing). Primary (readmission) and secondary clinical outcomes (ED visit, clinic attendance) did not require participant responses, so all participants were included in intent-to-treat analysis. Post-hoc, relative risks for readmissions using a *per-protocol analysis* and using *alcohol-related* readmissions were explored. Alcohol-relatedness was defined as alcohol consumption likely contributing to the indication for admission, as documented by the primary hospital team’s discharge summary. When readmission encounter details were unavailable, the readmission was considered alcohol-related by default. Due to small sample size, when available, partial data were included in the appendices (acceptability, adverse events, etc.). Data were analyzed in Python (version 3.7.6).

**Table 1:** Participant characteristics

	Arm 1 KET n = 13	Arm 2 NTX n = 14	Arm 3 LA n = 17	All Arms n = 44
Age (mean, sd)	43.92 (11.48)	44.93 (12.52)	46.17 (9.53)	45.11 (10.90)
*Gender, n (% of column)*				
Female	4 (30.8)	3 (21.4)	2 (11.8)	9 (20.5)
Male	9 (69.2)	11 (78.6)	15 (88.2)	35 (79.6)
Other Response	0 (0.0)	0 (0.0)	0 (0.0)	0 (0.0)
*Race, n (% of column)*				
White/Caucasian	9 (69.2)	4 (28.6)	12 (70.6)	25 (56.8)
Black/African American	0	2 (14.3)	1 (5.9)	3 (6.8)
American Indian/Alaska Native	1 (7.7)	4 (28.6)	2 (11.8)	7 (15.9)
Multiple/Other	3 (23.1)	4 (28.6)	2 (14.3)	9 (20.5)
*Ethnicity, n (% of column)*				
Non-Hispanic/Latinx	6 (61.5)	8 (57.1)	12 (70.6)	28 (63.6)
Hispanic/Latinx	4 (30.8)	6 (42.9)	5 (29.4)	15 (34.1)
Unknown / Other	1 (7.7)	0 (0.0)	0 (0.0)	1 (2.3)
*Housing Status, n (% of column)*				
Stable housing	9 (69.2)	10 (71.4)	8 (47.1)	27 (61.4)
Not stably housed / unknown	4 (30.8)	4 (28.6)	9 (52.9)	17 (38.6)
*Highest education completed, n (% of column)*				
No high school	1 (7.7)	2 (14.3)	2 (11.8)	5 (11.4)
High school / GED	6 (46.2)	9 (64.3)	9 (64.3)	24 (54.6)
Other/additional degree	6 (46.2)	3 (21.4)	6 (35.3)	15 (34.1)
*Family History of AUD, n (% of column)*				
1 + family members	9 (69.2)	12 (85.7)	15 (88.2)	36 (81.8)
2 + family members	6 (46.2)	4 (28.6)	7 (41.2)	17 (38.6)
*Past-year care utilization (mean, sd)*				
ED visits	8.46 (7.78)	9.64 (7.62)	13.82 (8.76)	10.91 (8.29)
Hospital admits	2.77 (2.59)	2.86 (2.80)	3.88 (5.33)	3.23 (3.88)
*Baseline behavioral / psychological characteristics (mean, sd)*				
Typical daily drinks	9.15 (7.04)	14.68 (11.52)	12.13 (9.86)	12.0 (9.69)
PHQ-9 score	14.08 (7.30)	13.50 (5.71)	13.18 (6.42)	13.55 (6.34)
ACE score	3.85 (2.34)	4.41 (3.07)	4.21 (2.65)	4.18 (2.65)
*Diagnoses among top 3 during index admission, n (%)*				
Neurological / Intoxication / Withdrawal	12 (92.3)	12 (85.7)	15 (88.2)	39 (88.6)
Infection / Sepsis	1 (7.7)	2 (14.3)	2 (11.8)	5 (11.3)
Gastrointestinal	5 (38.5)	3 (21.4)	4 (23.5)	12 (27.3)
Cardiopulmonary	4 (30.8)	4 (28.6)	6 (35.3)	14 (31.8)
Electrolytes / renal	5 (38.5)	5 (35.7)	3 (17.6)	13 (29.5)
Musculoskeletal	0 (0.0)	1 (7.1)	1 (5.9)	2 (4.5)
Psychiatric	0 (0.0)	0 (0.0)	1 (5.9)	1 (2.4)

**Table 2:** Primary and secondary clinical outcomes assessed by EHR query

		Arm 1 KET n = 13	Arm 2 NTX n = 14	Arm 3 LA n = 17
*30-day hospital readmission (binary)*				
Count (%)		2 (15.38%)	3 (21.43%)	7 (41.18%)
RR to LA arm		0.3736	.5204	
		p = 0.17	p = 0.27	
% of readmissions considered alcohol-related (post-hoc)		50.0%	100.0%	100.0%
*30-day ED visit (binary)*				
Rate (%)		7 (53.85%)	8 (57.14%)	12 (70.59%)
RR to LA arm		.7629	.8095	
		p = 0.58	p = 0.69	
*14-day clinic attendance (binary)*				
Rate (%)		8 (61.54%)	7 (50.00%)	7 (41.18%)
RR to LA arm		1.4944	1.2142	
		p = 0.46	p = 0.90	

## Results

### Recruitment and protocol feasibility

**Fig. 1:**
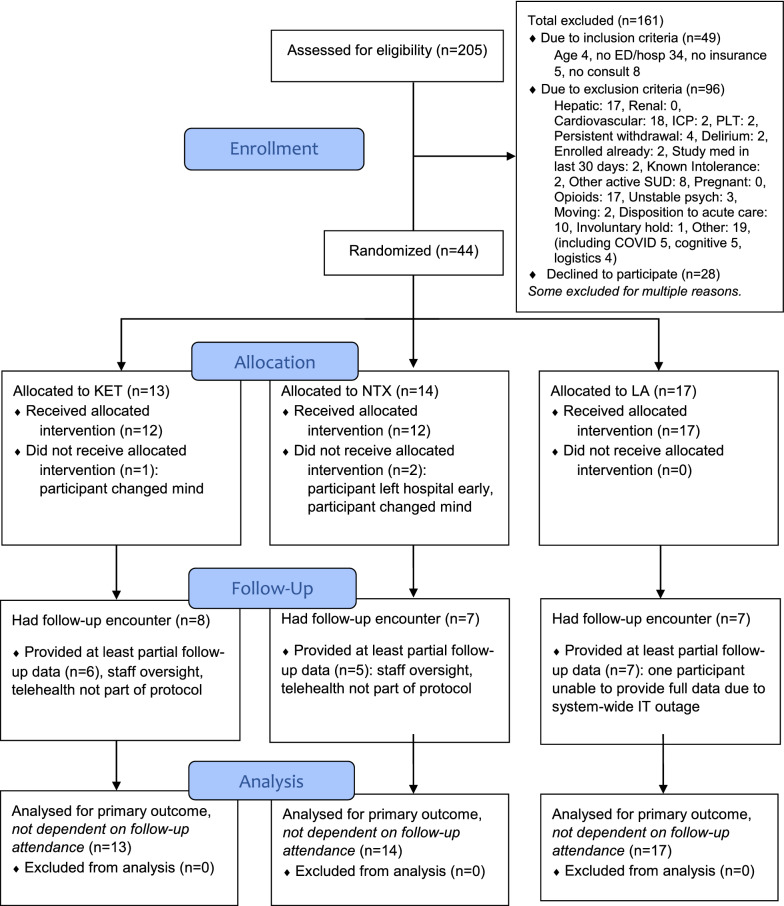
CONSORT [41] diagram

Of 205 patients assessed for eligibility, 28 declined (irrespective of eligibility), 49 did not meet all inclusion criteria (irrespective of interest), 96 met exclusion criteria (irrespective of interest), and 44 were ultimately consented and randomized (n = 13,14,17 for KET, NTX, LA arms, respectively) for intent-to-treat analysis (Fig. 1).

The average eligibility assessment rate was 17.1 patients per month. The average monthly recruitment rate was 3.7, resulting in a recruitment likelihood of 21.6%. Common reasons for ineligibility were having no prior hospital or ED visit (n = 34), having significant cardiovascular disease (n = 18), having significant hepatic disease (n = 17), using or anticipating use of opioids (n = 17), and having inappropriate post-hospital disposition (n = 10). Notably, 40 instances of study exclusion (Fig. 1) were specific to naltrexone (opioids, hepatic, platelets, prior recent receipt, and known intolerability); 20 were specific to ketamine (cardiovascular and intracranial pressure).

A majority of participants were non-Hispanic (63.6%), white/Caucasian (56.8%), males (79.6%) whose highest level of education was high school/GED (54.6%). A substantial portion did not have stable housing (38.6%). Clinically, they had a mean of 10.9 past-year ED visits and 3.2 hospital admissions, had a mean of 12.0 daily drinks at baseline, and had a neurologic (including intoxication/withdrawal) diagnosis among the top three encounter diagnoses 88.6% of the time (Table 1).

There were seven significant inpatient protocol deviations, each in separate participants (15.9% of total sample): participant did not receive assigned pharmacologic intervention (n = 2 in NTX arm, n = 1 in KET arm), participant received intervention in incorrect manner (n = 1), full clinic intake not performed prior to discharge (n = 3).

### Primary clinical outcome

For the KET, NTX, and LA arms, the thirty-day readmission rates were 15.4, 21.4, and 41.2%, respectively. Comparing the KET arm to the LA arm, the relative risk of readmission was 0.37 (p = 0.17) (Table 2). Comparing the NTX arm to the LA arm, the relative risk of readmission was 0.52 (p = 0.27). Readmissions (first instance if there were multiple) occurred on average at 16.9 days (S.D. 9.0) post-discharge.

Results did not vary substantially when using a post-hoc, per-protocol analysis (i.e., switching the three who did not receive their assigned pharmacologic intervention to the LA arm). When analyzing *alcohol-related* readmissions post-hoc, only one readmission event (in KET arm) was confirmed to be *not* alcohol-related, resulting in a relative risk of 0.19 for KET in relation to the LA arm. One readmission in the NTX arm lacked information to assess alcohol-relatedness.

### Secondary clinical outcomes

For the KET, NTX, and LA arms, thirty-day ED visit rates were 53.9, 57.1, and 70.6%, respectively. Fourteen-day addiction clinic attendance rates were 61.5, 50.0, and 41.2%, respectively. The hospital readmission rate among those who attended follow-up clinic was 22.7% compared to 31.9% among those who did not present to clinic (RR 0.71, p = 0.50).

Some data elements were re-assessed at the addiction clinic follow-up visit (including PHQ9, TLFB, and acceptability). Unfortunately, the attendance rate of all arms (n = 6,5,7 for KET, NTX, LA arms, respectively) precluded a robust comparison of findings.

### Acceptability and safety

Recorded immediately post-administration, mean acceptability scores of the KET and NTX interventions were 9.50 and 9.17 (Likert scale range 0–10), respectively, and mean anticipated effectiveness scores were 8.75 and 7.75, respectively.

Data recorded immediately post-administration (see Appendix) revealed no repeated instances of treatment-emergent (i.e., not present pre-administration) symptoms. Those that presented to follow-up and provided adverse event data completed the PRISE questionnaire (see Appendix). In this KET subset (n = 6), the most reported *distressing* symptoms since hospital discharge were shortness of breath (33.3%), anxiety (33.3%), poor concentration (33.3%), fatigue (33.3%), and restlessness (33.3%). In this NTX subset (n = 5), the most reported *distressing* symptoms since hospital discharge were shortness of breath (40.0%) and anxiety (80.0%). In this LA subset (n = 6), the most reported *distressing* symptoms since hospital discharge were tremor (33.3%) and blurred vision (33.3%). No serious adverse events occurred, although one participant mistakenly received intramuscular naltrexone in an upper extremity, with no ill effect after an extended period of observation. This participant was still included in analysis (intent-to-treat).

Vital signs remained stable among all arms, except for ketamine with a rise in systolic (mean + 12.66 mmHg, p = 0.006) and diastolic blood pressure (mean + 10.34 mmHg, p = 0.05) at 40 min. Both parameter changes attenuated at 160 min. There were no instances of treatment-emergent symptoms attributed to these vital sign changes. There were no known instances of participants using ketamine after hospital discharge.

Regarding dissociative symptoms, the mean CADSS score in the KET arm was 20.31 (standard deviation 16.62), nearly identical to that seen in a similar trial [42, 43].

## Discussion

In this small, randomized trial conducted in the hospital setting, we found that two single-dose, adherence-independent interventions were feasible to administer among a high-utilization AUD population. The IV ketamine and IM naltrexone arms had lower—albeit not statistically significant—rates of hospital readmission, ED presentation, and addiction clinic non-attendance. Despite randomization, the linkage alone arm appeared to be more male, have higher baseline hospital utilization, and be less stably housed than other arms. And the ketamine arm appeared to have fewer baseline drinks compared to other arms. These factors may have biased in favor of pharmacologic intervention. However, arms had similar mean depressive symptom scores and mean early life trauma scores, independent predictors of hospital utilization, respectively [35, 44].

While limited in generalizability, these data on ketamine are consistent with recent pre-clinical and clinical literature on ketamine’s use for AUD. In a human laboratory setting with non-treatment seeking, non-depressed adults with problematic drinking [17], ketamine reduced craving and drinking quantity at 10 days post-infusion to a greater extent than placebo. In a pilot randomized trial (n = 40) by Dakwar et al., non-depressed patients with AUD who received a single ketamine infusion (0.71 mg/kg over 52 min) showed a higher rate of no heavy drinking days (82% vs 59%) and attendance at a 21-day counseling visit (100% vs 75%) compared to the active control (midazolam) arm [42], suggesting the anti-craving effect of ketamine persists well beyond its detectable presence in circulation. And in a phase 2 clinical trial, Grabski et al. demonstrated that a series of 3 weekly infusions (0.8 mg/kg over 40 min) resulted in a reduced number of drinking days at 6 months compared to placebo, most pronounced when combined with relapse-prevention psychotherapy [45]. It is possible that our use of 0.5 mg/kg—common among trials for treatment resistant depression [14, 28]—was suboptimal in balancing potential efficacy with risk of distressing or dangerous side effects. However, as the dose approaches ranges considered “anesthetic,” infusions may require more comprehensive monitoring which may limit future availability outside of hospital settings.

Our results add to the growing data on IM naltrexone, which have been equivocal in support of use in hospital settings. One meta-analysis showed a trend toward fewer admissions compared to PO naltrexone [46]. However, one pilot study [25] (n = 54 randomized) conducted at a Veterans Affairs hospital with a majority admitted for a psychiatric indication, and one recently concluded clinical trial [47] (n = 248 randomized) at a safety-net hospital in Boston directly compared IM to PO naltrexone for AUD. Neither showed a clear benefit in terms of inpatient utilization, but Busch et al. reported a higher 3-month treatment retention among the IM naltrexone arm [25], and Saitz et al. reported a larger decrease in percent heavy drinking days for IM naltrexone [47]. Our positive results may suggest that IM naltrexone has an especially important role among the patients we targeted: those with baseline high hospital utilization.

Participants in our study rated ketamine infusions and naltrexone injections as highly acceptable, and those in the ketamine arm appeared to have the highest “expectancy” of effectiveness. Blinding would help to mitigate this bias, though trials using ketamine are difficult to effectively blind due to its unique psychoactive effects [42, 45]. We found it possible but challenging to recruit appropriate participants due to many exclusion criteria, particularly related to naltrexone safety (mostly related to hepatic disease and opioids). This, in addition to our readmission results, bolsters the case for focusing research on IV ketamine, which may have broader potential reach among inpatients with AUD.

### Strengths

Patients underwent simple randomization, reducing unmeasured confounding in the assignment of participants. To date, this may be the only study administering ketamine for AUD (i.e., not specifically for alcohol withdrawal [48]) among medical inpatients. It was conducted in a very pragmatic setting, generating data and lessons pertaining to real-world effectiveness. The patient population was characterized by dramatically high hospital utilization, allowing current and future investigation to inform potentially significant reductions in system costs and patient morbidity.

### Limitations

This open-label pilot trial demonstrated feasibility and estimated the effect sizes of two pharmacologic interventions. As such, it was under-powered for detecting statistically significant differences, especially comparing the two pharmacologic arms to each other. There was no blinding in this study, possibly resulting in strong expectancy effects with the pharmacologic interventions. Furthermore, this study did not include oral naltrexone as a comparator, which some advocate in favor of being standardized on hospital discharge [8]. The sample size required to detect the marginal benefit of IM vs PO naltrexone would have been beyond the scope of this pilot study.

While our follow-up rates were higher than previously achieved among our patient population (12% by one internal estimate), none of our study arms had follow-up rates high enough to make robust inferences regarding changes in drinking or other self-reported outcomes. Future investigation could explore methods to obtain these data utilizing substantial locator details obtained prior to discharge, enhanced incentives, and engagement in community settings [49].

## Conclusions

This pilot study demonstrated the feasibility of IV ketamine and verified the feasibility of IM naltrexone given pre-discharge in the medical hospital setting. Both single-dose interventions—not reliant on daily medication adherence in the immediate post-hospital period—had lower rates of all-cause, hospital readmission and higher rates of addiction clinic attendance than the enhanced-linkage control intervention, though differences were non-significant. Further investigation of this type of intervention given pre-discharge should be tested with blinding and with more robust follow-up data collected. If ultimately deemed successful, these interventions have the potential to change the standard of care for in-hospital AUD recovery initiation.

## Data Availability

The datasets used and/or analyzed during the current study are available from the corresponding author on reasonable request.

## Appendix

See Tables 3, 4, 5, 6.

**Table 3:** Acceptability

	Arm 1 KET		Arm 2 NTX		Arm 3 LA	
	Int (n = 12)*	FU (n = 6)	Int (n = 12)**	FU (n = 5)	Int (n = 17)	FU (n = 6)***
Acceptability of intervention, Likert 1–10: mean (sd)	9.5 (0.80)	9.5 (1.22)	9.17 (1.40)	8.67 (2.13)		
Effectiveness of intervention, Likert 1–10: mean (sd)	8.75 (1.48)	7.67 (3.61)	7.75 (2.34)	7.5 (2.06)		
Motivation of financial incentive, Likert 1–10: mean (sd)	2.42 (2.84)	4.00 (3.35)	4.46 (3.40)	4.17 (3.08)	2.5 (2.76)	4.33 (3.67

*1 participant did not receive intervention

**2 participants did not receive intervention

***7 participants provided some data, only 6 provided data listed in this table

**Table 4:** Vital signs

Treatment arm	Baseline SBP (mean mmHg)	Baseline DBP (mean mmHg)	Baseline HR (mean BPM)	40 min SBP (mean mmHg)	40 min DBP (mean mmHg)	40 min HR (mean BPM)	160 min SBP (mean mmHg)	160 min DBP (mean mmHg)	160 min HR (mean mmHg)
KET	125.17	83.83	88.33	137.83**	94.17*	91.00	134.45	85.36	93.27
NTX	126.23	80.85	88.54	127.0	83.08	89.33			
LA	126.71	86.12	87.76						

*p < 0.05, **p < 0.01 for H_0_ = no change from baseline

**Table 5:** Treatment-emergent adverse events reported immediately post-intervention. CADSS = clinician administered dissociative states scale

	Arm 1 NTX (n = 12)		Arm 2 KET (n = 12)	
	Tolerable n (%)	Distressing n (%)	Tolerable n (%)	Distressing n (%)
Lightheadedness				1 (8.3%)
Fatigue	1 (8.3%)			
Arm pain	1 (8.3%)		1 (8.3%)	
Nausea			1 (8.3%)	
Floating			1 (8.3%)	
Hot flashes	1 (8.3%)			
Dry mouth	1 (8.3%)			
Tingling	1 (8.3%)			
Body tightness			1 (8.3%)	
Lip numbness				1 (8.3%)
Blurred vision			1 (8.3%)	
Tingling			1 (8.3%)	
CADSS Score (mean, sd)			20.31 (16.62)	

**Table 6:** Adverse events since discharge reported at follow-up visit

	Arm 1 KET (n = 6 that gave these data)		Arm 2 NTX (n = 5 that gave these data)		Arm 3 LA (n = 6 that gave these data)	
	Tolerable n (%)	Distress n (%)	Tolerable n (%)	Distress n (%)	Tolerable n (%)	Distress n (%)
*Gastrointestinal*						
Diarrhea	1 (16.7)	1 (16.7)	0 (0.0)	1 (20.0)	1 (16.7)	1 (16.7)
Constipation	2 (33.3)	0 (0.0)	1 (20.0)	0 (0.0)	0 (0.0)	0 (0.0)
Dry Mouth	2 (33.3)	0 (0.0)	1 (20.0)	1 (20.0)	2 (33.3)	0 (0.0)
Nausea/vomiting	1 (16.7)	0 (0.0)	1 (20.0)	1 (20.0)	1 (16.7)	0 (0.0)
Abd pain	1 (16.7)	0 (0.0)	4 (80.0)	0 (0.0)	0 (0.0)	1 (16.7)
Incr appetite	3 (50.0)	0 (0.0)	3 (60.0)	0 (0.0)	2 (33.3)	0 (0.0)
Decr appetite	0 (0.0)	0 (0.0)	4 (80.0)	0 (0.0)	0 (0.0)	1 (16.7)
*Heart*						
Palpitations	1 (16.7)	0 (0.0)	1 (20.0)	1 (20.0)	1 (16.7)	0 (0.0)
Dizziness on standing	3 (50.0)	0 (0.0)	2 (40.0)	1 (20.0)	3 (50.0)	0 (0.0)
Chest pain	1 (16.7)	0 (0.0)	2 (40.0)	1 (20.0)	0 (0.0)	0 (0.0)
*Lungs*						
Shortness of breath	0 (0.0)	2 (33.3)	1 (20.0)	2 (40.0)	1 (16.7)	0 (0.0)
Cough	1 (16.7)	0 (0.0)	2 (40.0)	0 (0.0)	0 (0.0)	1 (16.7)
Pain with breathing	1 (16.7)	0 (0.0)	2 (40.0)	0 (0.0)	0 (0.0)	0 (0.0)
*Skin*						
Injection-site reaction (NTX-only)	0 (0.0)	0 (0.0)	0 (0.0)	0 (0.0)	0 (0.0)	0 (0.0)
Rash	0 (0.0)	0 (0.0)	0 (0.0)	0 (0.0)	0 (0.0)	0 (0.0)
Increased perspiration	1 (16.7)	0 (0.0)	1 (20.0)	0 (0.0)	0 (0.0)	1 (16.7)
Itching	0 (0.0)	0 (0.0)	1 (20.0)	0 (0.0)	1 (16.7)	0 (0.0)
Dry Skin	1 (16.7)	1 (16.7)	3 (60.0)	1 (20.0)	3 (50.0)	0 (0.0)
*Nervous system*						
Headache	1 (16.7)	0 (0.0)	2 (40.0)	1 (20.0)	1 (16.7)	0 (0.0)
Tremor	0 (0.0)	1 (16.7)	0 (0.0)	1 (20.0)	2 (33.3)	2 (33.3)
Poor coordination	3 (50.0)	0 (0.0)	2 (40.0)	1 (20.0)	3 (50.0)	0 (0.0)
Dizziness or lightheadedness	2 (33.3)	0 (0.0)	3 (60.0)	0 (0.0)	2 (33.3)	0 (0.0)
Numbness or tingling	1 (16.7)	0 (0.0)	2 (40.0)	0 (0.0)	2 (33.3)	0 (0.0)
Speech difficulty	0 (0.0)	0 (0.0)	2 (40.0)	1 (20.0)	0 (0.0)	0 (0.0)
*Eyes/ears*						
Blurred vision	2 (33.3)	0 (0.0)	1 (20.0)	1 (20.0)	2 (33.3)	2 (33.3)
Ringing in ears	1 (16.7)	0 (0.0)	0 (0.0)	0 (0.0)	1 (16.7)	0 (0.0)
*Genital/urinary*						
Difficulty urinating	0 (0.0)	0 (0.0)	0 (0.0)	0 (0.0)	2 (33.3)	0 (0.0)
Painful urination	0 (0.0)	0 (0.0)	0 (0.0)	0 (0.0)	0 (0.0)	0 (0.0)
Frequent urination	3 (50.0)	0 (0.0)	1 (20.0)	0 (0.0)	3 5(0.0)	0 (0.0)
Menstrual irregularity	0 (0.0)	0 (0.0)	0 (0.0)	0 (0.0)	0 (0.0)	0 (0.0)
Increased libido	2 (33.3)	0 (0.0)	0 (0.0)	0 (0.0)	0 (0.0)	0 (0.0)
Decreased libido	1 (16.7)	1 (16.7)	1 (20.0)	0 (0.0)	1 (16.7)	0 (0.0)
*Sleep*						
Difficulty sleeping	3 (50.0)	1 (16.7)	2 (40.0)	1 (20.0)	3 5(0.0)	0 (0.0)
Sleeping too much	1 (16.7)	0 (0.0)	0 (0.0)	1 (20.0)	1 (16.7)	0 (0.0)
Abnormal dreams	4 (66.7)	0 (0.0)	3 (60.0)	1 (20.0)	1 (16.7)	0 (0.0)
*Other*						
Anxiety	3 (50.0)	2 (33.3)	2 (40.0)	4 (80.0)	4 (66.7)	1 (16.7)
Hallucinations	1 (16.7)	0 (0.0)	1 (20.0)	0 (0.0)	1 (16.7)	1 (16.7)
Memory Loss	2 (33.3)	1 (16.7)	2 (40.0)	1 (20.0)	0 (0.0)	1 (16.7)
Poor concentration	1 (16.7)	2 (33.3)	3 (60.0)	1 (20.0)	3 (50.0)	0 (0.0)
Fatigue or decreased energy	0 (0.0)	2 (33.3)	4 (80.0)	1 (20.0)	3 (50.0)	0 (0.0)
Restlessness	2 (33.3)	2 (33.3)	4 (80.0)	1 (20.0)	0 (0.0)	0 (0.0)
Other	0 (0.0)	0 (0.0)	1 (20.0)	0 (0.0)	0 (0.0)	0 (0.0)
